# Platelet-released growth factors promote pro-angiogenic effects in endothelial cells: a translational perspective on wound healing and regenerative medicine

**DOI:** 10.1186/s13287-026-05214-y

**Published:** 2026-08-05

**Authors:** Michael Singh, Karina Zitta, Thilo Wedel, Ralph Lucius, François Cossais, Peter Behrendt, Sabine Fuchs, Jan-Tobias Weitkamp, Rouven Berndt, Olga Zimmermann, Tim Schricker, Andreas Bayer, Martin Albrecht

**Affiliations:** 1 Institute of Anatomy, Kiel University, Otto-Hahn-Platz 8, D - 24118 Kiel, Germany; 2 Department of Anesthesiology and Intensive Care Medicine, University Hospital Schleswig-Holstein, Kiel, Germany; 3 Experimental Trauma Surgery, Department of Orthopedics and Trauma Surgery, University Medical Center Schleswig-Holstein, Kiel, Germany; 4 Department of Oral and Maxillofacial Surgery, University Medical Center Schleswig-Holstein, Kiel, Germany; 5 Clinic of Vascular Medicine, University Heart & Vascular Center Hamburg-Eppendorf, Hamburg, Germany; 6 Department of Neurology, University Medical Center Schleswig-Holstein, Kiel, Germany; 7 University Clinic of Dermatology and Allergy, Klinikum Oldenburg, Oldenburg, Germany; 8 Univ Rouen Normandie, INSERM, Normandie Univ, ADEN UMR1073 “Nutrition Inflammation and Microbiota-Gut-Brain Axis”, Rouen, France

**Keywords:** Platelet-released growth factors (PRGF), Human endothelial cells, Angiogenesis

## Abstract

Platelet-Released Growth Factors (PRGF) are a standardized, cell- and fibrin-free platelet lysate with translational potential for tissue repair. PRGF enhance tissue regeneration, particularly in contexts where angiogenesis is critical for restoring perfusion in chronic and non-healing wounds. Here, we present the first in vitro study demonstrating that PRGF exert pro-angiogenic effects at a working concentration of 10%. The study employs an endothelial cell-based model integrating targeted gene expression analysis, array-based profiling of angiogenesis-related proteins, and functional endothelial tube formation assays. In this setting, screening-level transcriptional and proteomic profiling indicated higher transcript levels of angiogenesis-related genes and increased signals of representative angiogenesis-associated mediators (e.g., Platelet Factor 4, Angiopoietin-1, Angiogenin) following PRGF stimulation. Functional tube formation assays demonstrated significant enhancement of multiple pro-angiogenic parameters in response to PRGF, including number of pieces (P < 0.001), junctions (P < 0.01), and segments (P < 0.05). These findings suggest that 10% PRGF may support angiogenesis and tissue regeneration and provide a foundation for further validation in more complex multicellular systems and in vivo models.

**Supplementary Information:**

The online version contains supplementary material available at 10.1186/s13287-026-05214-y.

## Background

Chronic and complicated, non-healing wounds remain a major challenge across many clinical disciplines [1]. One key cause is inadequate vascularization, as seen in peripheral artery disease or diabetic ulcers [1], where the wound microenvironment is characterized by chronic inflammation and reduced angiogenic capacity [2]. Angiogenesis, the formation of new vessels from pre-existing ones, is essential for restoring oxygen and nutrient delivery, enabling immune cell access, and promoting wound closure and regenerative capacity [2, 3]. Angiogenesis in wounds is dominated by endothelial cells, with early activation during inflammation followed by sprouting and network formation in the proliferative phase [1, 3].

Platelet-derived products, which vary considerably in processing, formulation, and composition [4], are already employed in regenerative medicine [5]. Platelet-rich plasma (PRP) is an umbrella term for plasma-based platelet concentrates, typically generated through a two-step centrifugation process that enriches platelets within a defined plasma volume and yields a heterogeneous suspension of platelets, leukocytes, and plasma proteins [6]. Platelet-rich fibrin (PRF) a second-generation platelet concentrate is obtained by processing whole blood samples without anticoagulant, resulting in a polymerized three-dimensional fibrin matrix that serves as a growth-factor–rich scaffold supporting cell migration and proliferation [7]. PRP and PRF compositions are typically based on variable numbers of platelets and leukocytes, along with cytokines, signaling molecules, and structural proteins such as fibrin that can modulate immune responses and tissue repair [2, 4, 6, 8]. As a result heterogeneous clinical outcomes have been observed with PRP and PRF [9, 10] partly due to inconsistent terminology and inadequate characterization and reporting of leukocyte content and fibrin architecture.

In contrast, Platelet-Released Growth Factors (PRGF) represent a cell- and fibrin-free platelet lysate produced through defined mechanical and thermal lysis [11], yielding a reproducible, liquid formulation enriched in soluble platelet-derived mediators. Angiogenesis-related mediators encompass potent pro-angiogenic growth factors (e.g. Angiogenin, IL-8 [12]), vessel-stabilizing mediators (e.g. PDGF, Angiopoietins [13]), inflammatory chemokines (e.g. IL-8, PF4 [12]), and matrix-regulating proteases/inhibitors (e.g. Serpin familiy, TIMP [12]) that coordinate endothelial activation and morphogenesis and act predominantly during the inflammatory and proliferative phases of wound healing [8, 11]. Its liquid nature and controlled composition allow precise dosing and application, making PRGF suitable for a broad range of application-oriented settings, including those involving delicate or microvascular tissues.

Here, we focus on PRGF, a standardized, cell- and fibrin-free platelet lysate, and address the current gap in defining an effective working concentration using an established endothelial angiogenesis assay, complemented by screening-level molecular and proteomic profiling. The aim of this pilot study was to determine whether PRGF can directly induce pro-angiogenic responses in human endothelial cells and to identify an optimal concentration to guide future translational research.

## Methods

For more detailed information on the materials and methods used, please refer to the Supplementary Material files S1–S6.

### Preparation of PRGF and experimental setup

**Fig. 1:**
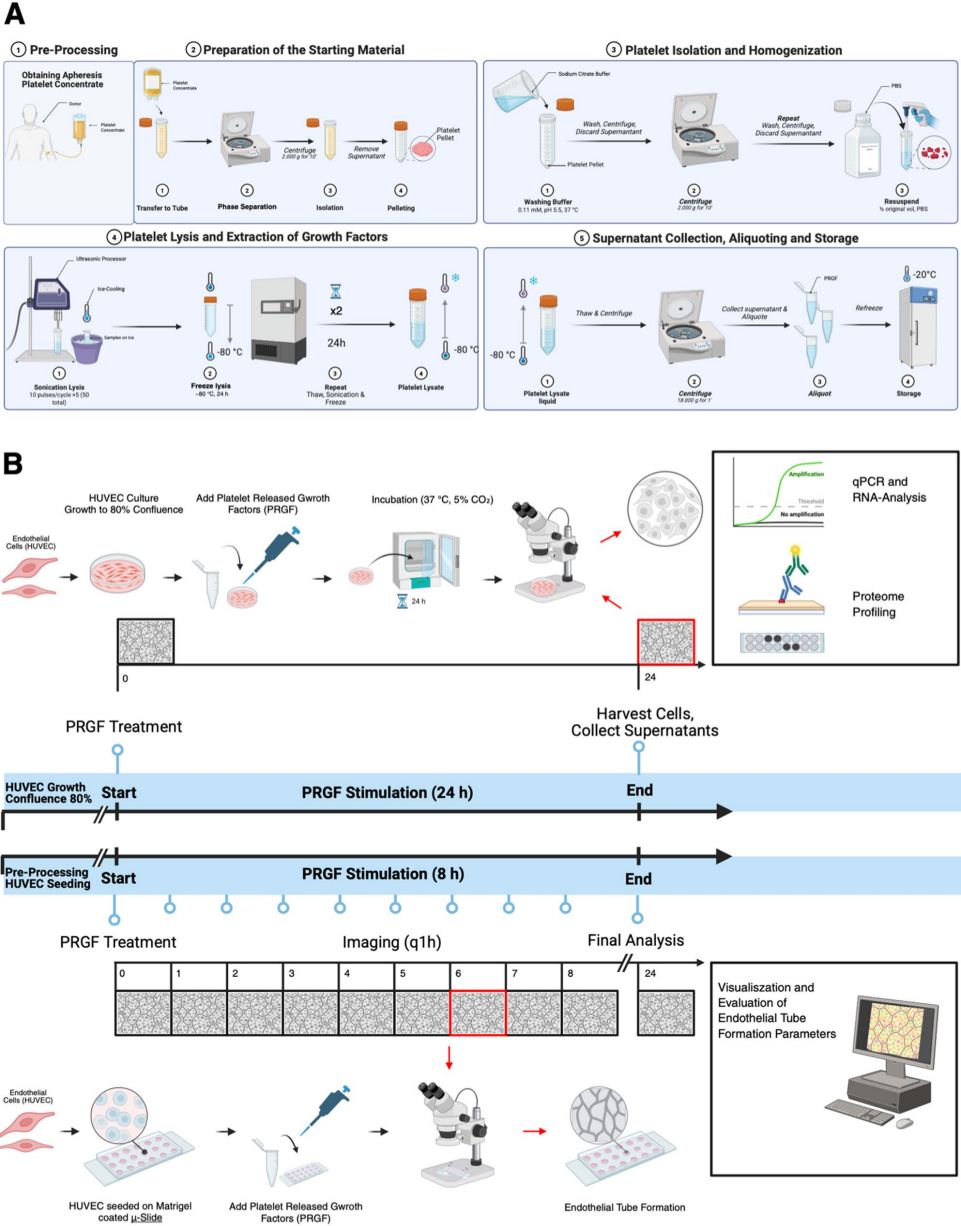
**A** PRGF Preparation. **B** Experimental Setup. **A** PRGF Preparation. Leukocyte-depleted platelet concentrates obtained from healthy donors were used as starting material. Following removal of the supernatant, the platelet pellet was resuspended and homogenized in phosphate-buffered saline (PBS). Platelet lysis was then induced by sequential mechanical ultrasonic disruption and thermal treatment, resulting in the generation of platelet-released growth factors (PRGF). **B** Experimental setup. Upper panel (molecular readouts): HUVECs were treated with PRGF at concentrations of 2%, 5%, and 10% starting at 80% confluence for a fixed period of 24 h. Gene expression analysis by qPCR and proteomic profiling were performed as complementary screening-level readouts from the same experimental setup. Lower panel (functional readout): The effects of PRGF (2%, 5%, and 10%) were assessed in a HUVEC tube formation assay. The red box indicates the time point selected for qualitative microscopic evaluation, followed by quantitative image-based analysis of angiogenic network formation. Created in BioRender https://BioRender.com/7zbppcz

All experiments were approved by the Ethics Committee of the University Medical Center Schleswig-Holstein (protocols D516/21, D519/18 and D518/13). PRGF was prepared from freshly donated leukocyte-depleted platelet concentrates as described previously [11, 14] and used as four donor-specific, non-pooled preparations in all experiments; pooling was limited to the pilot dataset in Supplementary Material S7. Briefly, each apheresis platelet concentrate contained an average concentration of 1.2 ± 0.4 × 10⁶ platelets/µl (range 0.44-2.0 × 10⁶ platelets/µl; PEI.H.03191.01.1). For experimental use, the defined starting materials (1 × 10⁶ platelets/µl) were processed following a standardized protocol [11]. PRGF preparation included repeated ultrasonic disruption and freeze–thaw lysis. Subsequent washing, resuspension and aliquoting steps, as well as further methodological details are illustrated in Fig. 1A (for details see also Supplementary Material S1).

### Culture and treatment of primary human endothelial cells with PRGF

Human umbilical vein endothelial cells (HUVEC) were isolated from umbilical cords and cultured as described before [15]. HUVEC (Passage 9) were maintained in endothelial cell growth medium with supplement (Promocell), and 10% fetal bovine serum (Thermo Fisher) at 37 °C and 5% CO₂. For stimulation experiments, cells were seeded in 12-well plates and cultured to 80% confluence, which served as the starting condition for PRGF treatment. Culture medium was replaced with fresh medium containing 0%, 2%, 5%, or 10% PRGF and cells were incubated for an additional 24 h. Following 24 h of stimulation, cells were processed for RNA extraction and the corresponding culture supernatants were collected and stored at − 20 °C for proteomic profiling (Fig. 1B). Because molecular and proteomic analyses were conducted at a single screening time point, the resulting readouts reflect acute responses and do not permit conclusions regarding sustained effects. For further details, see Supplementary Material S2.

### RNA isolation and quantitative real-time PCR

Total RNA was extracted using the RNeasy Mini Kit (Qiagen) according to the manufacturer’s specification and quantified spectrophotometrically. cDNA synthesis was performed following standard procedures (Supplementary Material S3) [11]. Quantitative real-time PCR was carried out using TB Green Premix Ex Taq II (Takara Bio) on a StepOne Plus system (Thermo Fisher). The qPCR panel was designed as a 24 h exploratory screening set to capture endothelial activation and inflammation-associated pro-angiogenic signaling. Gene expression of ANG-2, VCAM-1, ICAM-1, and IL-8 was normalized to RP38. Primer sequences are provided in Supplementary Material S4.

### Human angiogenesis proteome profiling array

Cell culture supernatants were analyzed for angiogenesis-related proteins using the Human Angiogenesis Proteome Profiler Array (R&D Systems) following the manufacturer’s instructions. Samples were pre-incubated with a detection antibody mix and applied to membranes carrying antibodies against 55 target proteins. Membranes were incubated, washed and developed by chemiluminescence. Signals were imaged using the Fusion FX Vilber (Vilber Lourmat) and quantified with ImageJ (NIH). Only proteins above 10% reference-spot intensity were further evaluated [12]. Assay performance was verified using internal reference and negative control spots. Two baseline (BL) reference profiles were processed in parallel under identical conditions: BL1 (ECGM, no cell contact) to capture culture-medium background signals, and BL2 (10% PRGF in PBS, no cell contact) to account for proteins intrinsically present in PRGF. The resulting profiles are interpreted as screening-level contextual data. Extended methodological details are provided in Supplementary Material S5.

### In vitro tube formation assay

Tube formation assays were performed in ibidi µ-Slide 15 Well chambered coverslips (ibidi GmbH) coated with Corning Matrigel^®^ Matrix. Matrigel was handled on ice and polymerized at 37 °C prior to cell seeding. HUVECs (10,000 cells/well) were seeded onto the Matrigel layer in ECGM and treated with PRGF at 2%, 5%, or 10%, while untreated cells served as controls. Cultures were maintained at 37 °C and 5% CO₂, and phase-contrast images were acquired every hour for 8 h, with an additional image captured at 24 h. Tube formation was quantified using the workflow for the Angiogenesis Analyzer plugin for ImageJ (NIH) [16] with identical analysis settings applied across all conditions. Readouts included morphometric parameters such as segments, junctions, pieces, and branches. Detailed assay procedures, parameter definitions, and analysis settings are provided in Supplementary Material S6 and online: http://image.bio.methods.free.fr/ImageJ/?Angiogenesis-Analyzer-for-ImageJ&lang=en.

### Statistics

All data are expressed as mean ± SD. Graphs were generated and statistical analyses performed with GraphPad Prism 8. Data were tested for normality (Kolmogorov–Smirnov test) and transformed if necessary. Tube formation group comparisons were analyzed by one-way ANOVA followed by Tukey’s multiple comparisons test. *p* < 0.05 was considered statistically significant.

## Results

### 10% PRGF modulates the expression and secretion of pro-angiogenic factors in endothelial cells

**Fig. 2:**
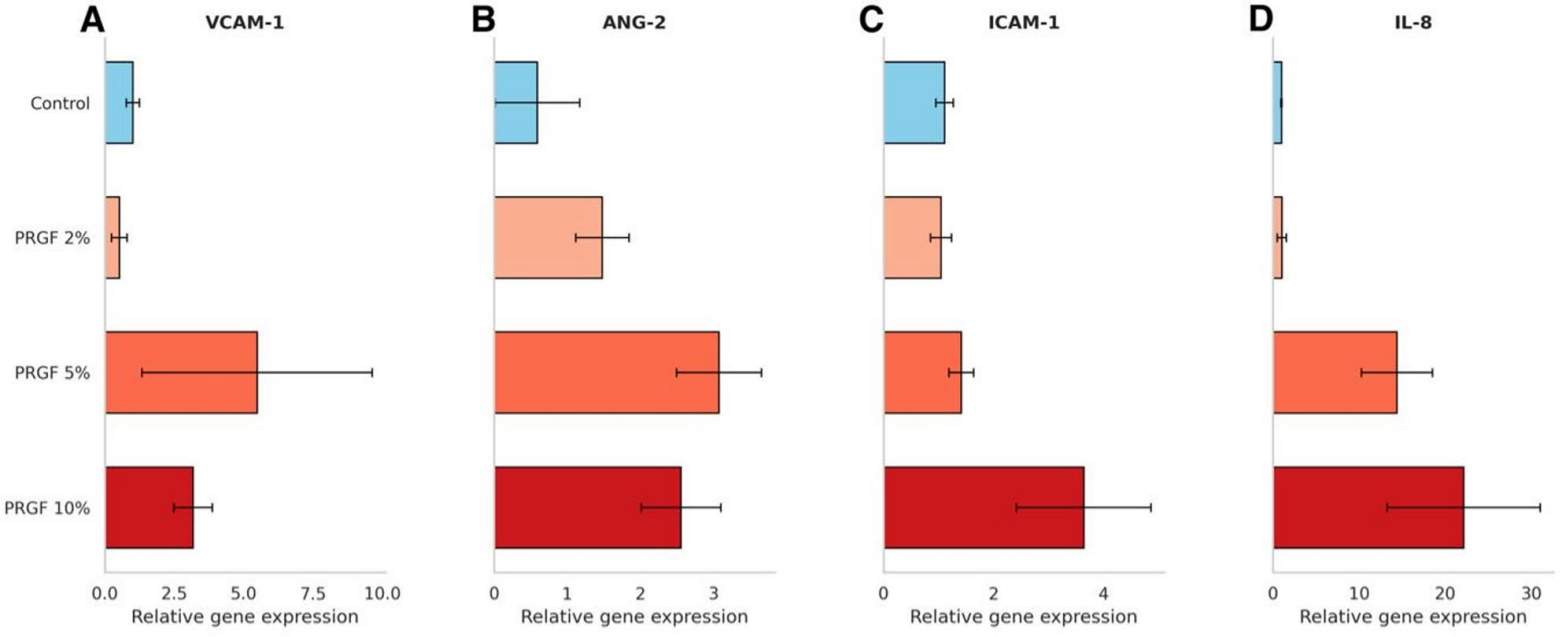
PRGF modulates angiogenesis-associated gene expression in endothelial cells. Relative expression levels of VCAM-1 (**A**), ANG-2 (**B**), ICAM-1 (**C**) and IL-8 (**D**) following treatment with PRGF. Data represent mean ± SD (control *n* = 2; PRGF groups *n* = 3). Data analysed with GraphPad 8

Gene expression analysis was performed to screen the effect of PRGF on angiogenesis-related genes VCAM-1 [17], ANG-2 [18], ICAM-1 [19] and IL-8 [20] in HUVEC. Cells were treated with control medium or different concentrations of PRGF for 24 h and gene expression was analyzed by qPCR. Given the limited sample size (*n* = 2–3), the results are reported as exploratory. Treatment with 5% and 10% PRGF was associated with increased transcript levels of angiogenesis-related genes (VCAM-1, ANG-2, ICAM-1, and IL-8; Fig. 2).

To assess PRGF-related changes at the protein level, proteomic profiling was performed with supernatants from HUVEC cultures treated with different concentrations of PRGF for 24 h.

**Fig. 3:**
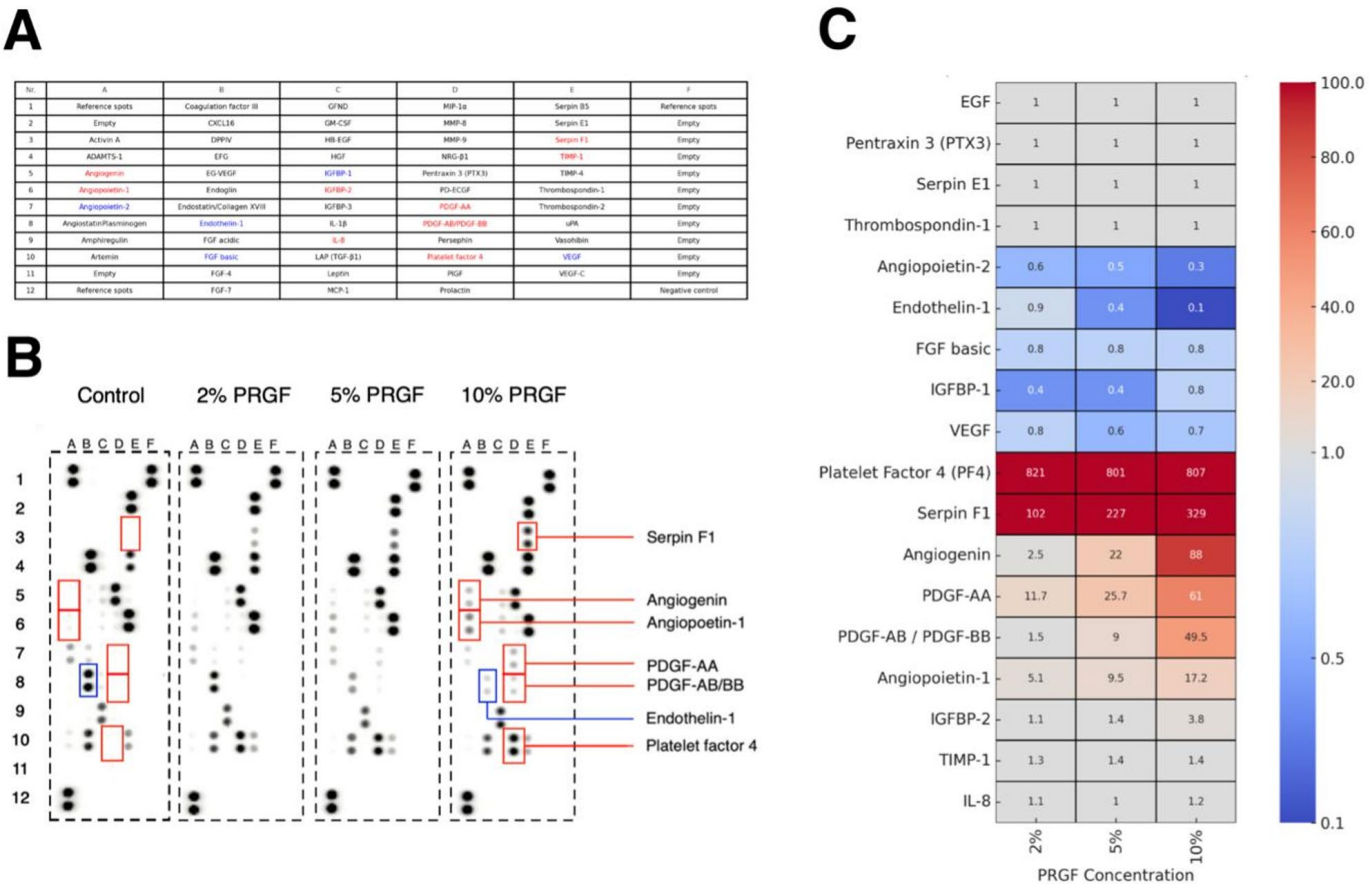
Effects of PRGF on the secretion of angiogenesis-related proteins by cultured endothelial cells. HUVEC were treated with PRGF for 24 h and protein secretion in cell culture supernatants was analyzed using the Human Angiogenesis Proteome Profiler Array. **A** Coordinates of the detected proteins on the array membrane **B** Representative array membranes showing signal intensities of 55 angiogenesis-related proteins with or without PRGF treatment. Proteins with changes ≥ 10-fold are highlighted. **C** Heatmap illustrating fold changes in protein secretion after PRGF treatment relative to control (= 1). Red shades indicate increased and blue shades decreased secretion levels. Array membranes were imaged with the Fusion FX System (Vilber Lourmat) and quantified with ImageJ (NIH)

Treatment with 10% PRGF modulated the secretory profile of approximately 25% (14 out of 55) of the analyzed angiogenesis-related proteins. Proteins with the highest increase in secretion were Platelet Factor 4 (807-fold), Serpin F1 (329-fold), Angiogenin (88-fold), PDGF-AA (61-fold), PDGF-AB/BB (49-fold) and Angiopoietin-1 (17-fold). In contrast, the secretion of several other factors (e.g. Endothelin-1) decreased slightly (Fig. 3). A complete list of all targets and underlying densitometry values are provided in Supplementary Tables S1 and S2.

### 10% PRGF leads to increased in vitro angiogenesis

**Fig. 4:**
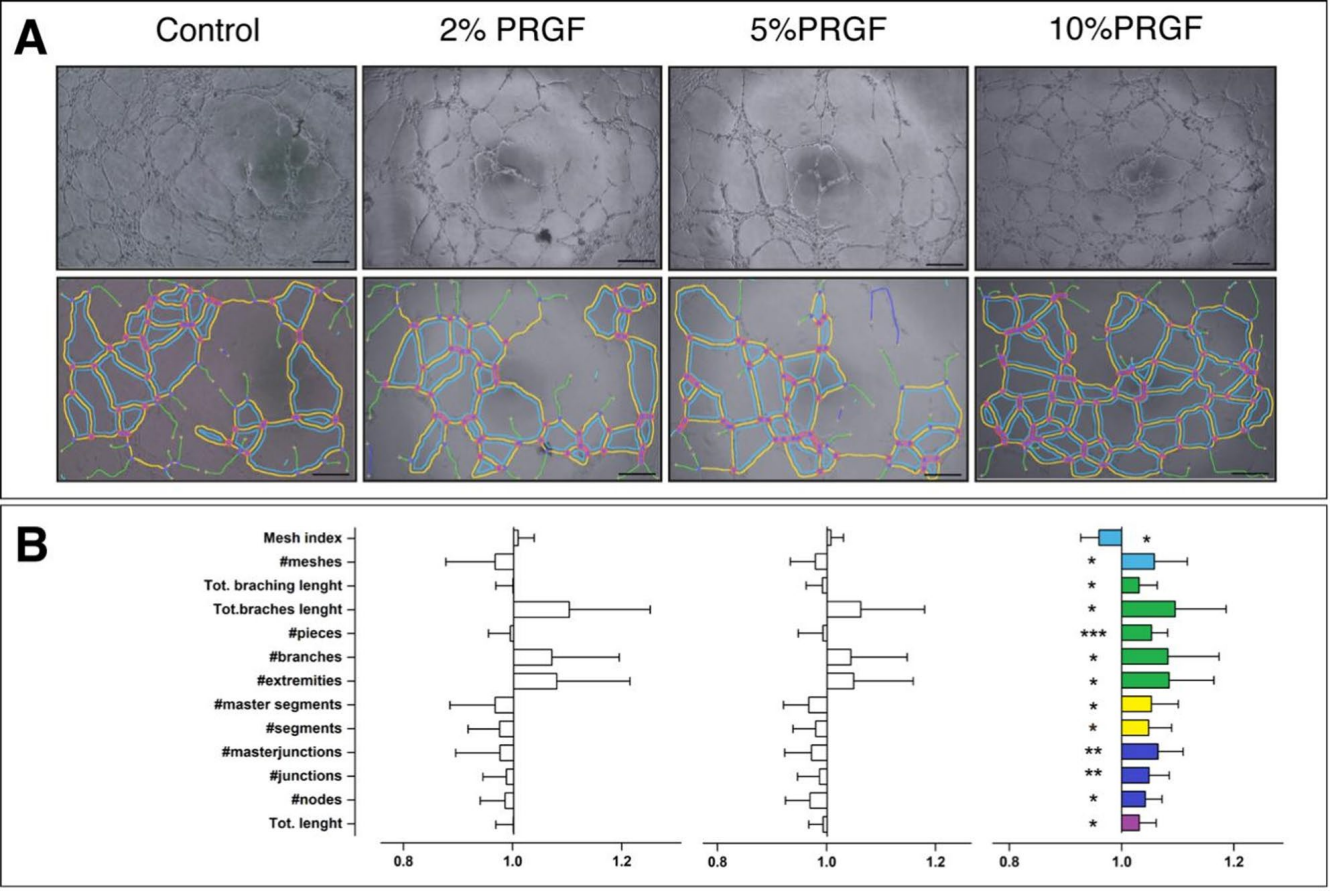
Influence of 10% PRGF on in vitro angiogenesis. HUVEC were cultured on Matrigel-coated surfaces and treated with PRGF or control medium. **A** Representative images showing tube formation in control and PRGF-treated cultures. Scale bars = 100 μm. **B** Quantitative analysis of tube formation parameters after PRGF treatment. Data represent mean ± SD of 9 independent experiments. *, *P* < 0.05; **, *P* < 0.01; ***, *P* < 0.001 (one-sample t-test vs. 1). Green = branches-related parameters; yellow = segment-related parameters ; cyan = meshes-related parameters; blue = nodes-related parameters; purple = length-related parameters. Network parameters were quantified using the ImageJ Angiogenesis Analyzer (Carpentier et al., Sci Rep 2020; doi:10.1038/s41598-020-67289-8)

To evaluate the translational angiogenic potential of PRGF, in vitro endothelial tube formation assays were performed using HUVEC cultured on Matrigel-coated dishes (Fig. 1B). This model mimics early steps of capillary formation in the contex of angiogenesis and provides translational insight into PRGF-induced vascular responses. Treatment with 10% PRGF promoted the formation of smaller, more continuous, and well-organized capillary-like networks compared with the control, with earlier mesh establishment and improved overall organization (Fig. 4A). In agreement with these qualitative observations, quantitative analysis revealed significant increases in parameters associated with early network organization, including number of pieces (*p* < 0.001), junctions (*p* < 0.01), segments (*p* < 0.05), and meshes (*p* < 0.05; Fig. 4B). Additional mesh-related network descriptors, including non-significant metrics (mean mesh size, tot. meshes area, branching interval, tot. isol. branches length, tot. segments length, nb isol. seg, and tot. master segments length) are reported in Supplementary Material S6.

## Discussion

Autologous platelet concentrates (APCs) are widely used across multiple medical disciplines due to their regenerative properties. APCs represent an established therapeutic approach for chronic and non-healing wounds, as well as in reconstructive, plastic, dermatologic, and orthopedic applications [21–23]. In contrast to platelet-rich plasma (PRP) and platelet-rich fibrin (PRF), platelet-rich growth factors (PRGF) provide a defined, cell-free and fibrin-free formulation enriched in soluble bioactive molecules [8, 11] making it a suitable agent for controlled tissue regeneration [10].

Angiogenesis is a prerequisite for effective tissue repair, as it restores perfusion to damaged or ischemic tissue, thereby enabling the delivery of oxygen, nutrients, and immune cells [2, 24].

Here, we present the first in vitro study demonstrating that PRGF exerts pro-angiogenic effects at a working concentration of 10%, supporting its potential role in tissue regeneration and related clinical applications.

Treatment with 10% PRGF significantly enhanced the formation of capillary-like structures in human endothelial cell cultures, promoting key steps of neovascularization in an established angiogenesis model [12, 25, 26]. Quantitative image analysis revealed increased junction formation, segment numbers, and overall network development, particularly at early time points (6 h), indicative of an accelerated pro-angiogenic response. While tube formation metrics capture distinct aspects of network organization and may vary depending on the time point assessed [27], 10% PRGF consistently induced the most robust pro-angiogenic phenotype characterized by enhanced early network organization and mesh establishment. However, it cannot be excluded that lower concentrations of PRGF exert effects at different kinetic phases that are not captured by this snapshot analysis.

To place this functional phenotype into a molecular context, transcriptional and proteomic readouts were assessed as screening-level descriptors. At the transcriptional level, PRGF stimulation was associated with increased expression of angiogenesis-related genes (VCAM-1, ANG-2, ICAM-1, and IL-8). Consistently, secretome profiling revealed increased signals for several endothelial/angiogenesis associated mediators, including Platelet Factor 4, Serpin F1, Angiogenin, PDGF-AA, PDGF-AB/BB, and Angiopoietin-1, collectively indicative of a pro-angiogenic and regulatory response.

However, the apparent decrease of some factors (e.g., Endothelin-1, Angiopoietin-2, VEGF) in our screening readouts likely reflects temporal dynamics, endothelial storage compartments, and feedback regulation in activated endothelial cells, as transcription, secretion, and structural morphogenesis do not necessarily peak simultaneously. Consistent with this interpretation, Angiopoietin-1 has been reported to suppress the release of Endothelin-1 from endothelial cells [28]. Structural and morphogenetic effects observed in the angiogenesis assay suggest that the pro-angiogenic activity of PRGF is not driven by individual factors alone, but rather by a balanced interplay of soluble growth factors and cytokines acting synergistically at the level of endothelial organization. In line with this concept, Angiopoietin-1/Tie2 signaling promotes the formation of well-branched HUVEC tube networks and increases network complexity, illustrating how individual components within a complex factor milieu shape the overall pro-angiogenic phenotype [32]. Nevertheless, given the limited coverage of the profiling panel, these molecular findings should be considered exploratory and hypothesis-generating rather than conclusive evidence of underlying mechanisms.

Corroborating the translational relevance of these findings, longer term pilot studies in outgrowth endothelial cell (OEC) mono-cultures and co-cultures with adipose tissue–derived mesenchymal stromal cells (ASC), serving as a wound healing model, independently provided supportive validation of the pro-angiogenic activity of PRGF in a more complex cellular context (Supplementary Material S7). These observations are further complemented by our previous studies demonstrating PRGF-induced wound healing programs in fibroblasts and ex vivo skin explants [29]. From a translational perspective, the liquid and fibrin-free nature of PRGF enables precise dosing and localized application, particularly in delicate or microvascular tissues where fibrin-based products may be less suitable. Together with our previous findings demonstrating improved epithelial barrier function, extracellular matrix formation and chondrocyte protection [11, 14, 29–34], the pro-angiogenic effects observed here support the potential of PRGF as a standardized, application-oriented candidate for further clinical evaluation in contexts such as chronic wounds, ischemic tissue injury, and anatomically restricted compartments, including the central nervous system and ocular microvasculature.

This study has several limitations. The use of HUVEC as a reference endothelial model, characterized by high baseline angiogenic activity, may influence mediator levels and morphometric outcomes. In addition, concentration-dependent feedback mechanisms and the temporal dynamics of transcriptional and secreted mediator responses across PRGF concentrations cannot be fully resolved within the current screening framework and warrant follow-up investigation. Finally, a limitation of this study is the lack of validation in additional wound-relevant cell types and in vivo models, which is necessary to assess relevance beyond the in vitro setting.

In summary, our findings demonstrate that PRGF promotes angiogenesis in vitro, with 10% PRGF emerging as the most effective concentration in our experimental setting. While direct effects on tissue regeneration were not assessed in this study, the observed pro-angiogenic activity provides a rationale for the potential role of PRGF in regenerative processes. Together, our results form a basis for further validation in more complex multicellular systems and in vivo models. Given its defined composition and practical availability, PRGF represents a promising candidate for translational approaches aimed at enhancing vascularization and tissue repair.

## Supplementary Information

Below is the link to the electronic supplementary material.

Supplementary Material 1.

Supplementary Material 2.

Supplementary Material 3.

Supplementary Material 4.

Supplementary Material 5.

Supplementary Material 6.

Supplementary Material 7.

Supplementary Material 8.

Supplementary Material 9.

## Data Availability

The datasets used during the current study are available from the corresponding author on reasonable request.

## Abbreviations

PRGF: Platelet-released growth factors
HUVEC: Human umbilical vein endothelial cells
OEC: Outgrowth endothelial cells
ASC: Adipose tissue derived mesenchymal stromal cells
ICAM-1: Intercellular adhesion molecule 1
VCAM-1: Vascular cell adhesion molecule 1
IL-8: Interleukin 8
ANG-2: Angiopoietin-2
qPCR: Quantitative polymerase chain reaction
RP38: Ribosomal protein 38
APCs: Autologous platelet concentrates
