# Supplementary material for: Platelet-released growth factors promote pro-angiogenic effects in endothelial cells: a translational perspective on wound healing and regenerative medicine
Source: Stem Cell Res Ther. 2026 Aug 5;17:270. doi: 10.1186/s13287-026-05214-y (PMC13445927; doi:10.1186/s13287-026-05214-y)
Supplement: Supplementary file 1 [file 13287_2026_5214_MOESM1_ESM.docx]

**Supplementary Materials S1**

Detailed PRGF preparation protocol:

PRGF was prepared from freshly donated leukocyte-depleted platelet concentrates as described previously [1,2]. Each leukocyte-depleted apheresis platelet concentrate contained an average concentration of 1.2 ± 0.4 × 10⁶ platelets/µL (range 0.44–2.0 × 10⁶ platelets/µL; PEI.H.03191.01.1). For experimental use, starting material was standardized to 1 × 10⁶ platelets/µL following a standardized protocol[1]. All donors provided written informed consent in accordance with approval of the Ethics Committee of the University Medical Centre Schleswig-Holstein, Kiel, Germany (protocol D516/21).

All platelet concentrates were obtained from the Institute of Transfusion Medicine, University Medical Centre Schleswig-Holstein, Campus Kiel, transported at 22 °C ± 2 °C, handled under sterile conditions (flow bench), and centrifuged for 10 min at 2.000 g. The resulting platelet pellet underwent two washing steps in sodium citrate buffer (0.11 mM, pH 5.5, 37 °C), each followed by centrifugation for 10 min at 2.000 g.

Platelets were resuspended in half the initial concentrate volume using PBS without Ca²⁺/Mg²⁺ (PAN-Biotech GmbH, Aidenbach, Germany) and maintained on ice. Mechanical lysis was performed using a Hielscher Ultrasonic processor (settings: P4W, C 80%, A 60%) with 10 ultrasonic impulses per cycle, followed by cooling on ice; this cycle was repeated five times (total 50 impulses). Thermal lysis was performed by freezing at −80 °C for 24 h. The suspension was thawed, subjected to the above mentioned ultrasonic procedure, and frozen again at −80 °C for another 24 h. After a final thaw, lysates were centrifuged for 1 min at 18.000 g to remove debris and small particles. The supernatant was collected as PRGF, aliquoted, and stored at −20 °C. Four independent PRGF preparations (PRGF1–PRGF4) from different donors (Donor1–Donor4) were used.

**References:**

1. Bayer A, Lammel J, Rademacher F, Groß J, Siggelkow M, Lippross S, et al. Platelet-released growth factors induce the antimicrobial peptide human beta-defensin-2 in primary keratinocytes. Exp Dermatol; 2016;25:460–5. https://doi.org/10.1111/EXD.12966

2. Bayer A, Lammel J, Tohidnezhad M, Lippross S, Behrendt P, Klüter T, et al. The Antimicrobial Peptide Human Beta-Defensin-3 Is Induced by Platelet-Released Growth Factors in Primary Keratinocytes. Mediators Inflamm; 2017;2017. https://doi.org/10.1155/2017/6157491
