# Supplementary material for: Platelet-released growth factors promote pro-angiogenic effects in endothelial cells: a translational perspective on wound healing and regenerative medicine
Source: Stem Cell Res Ther. 2026 Aug 5;17:270. doi: 10.1186/s13287-026-05214-y (PMC13445927; doi:10.1186/s13287-026-05214-y)
Supplement: Supplementary file 2 [file 13287_2026_5214_MOESM2_ESM.docx]

**Supplementary Table S2**

Signal values of target proteins under control conditions and PRGF stimulation:

| Coordinate | Target | Control | 2%PRGF1 | 5% PRGF1 | 10% PRGF1 |
| --- | --- | --- | --- | --- | --- |
| A1 | Ref spots upper left | 245.982 | 241.259 | 245.616 | 242.009 |
| A2 | Ref spots upper left | 242.25 | 245.893 | 246.357 | 248.098 |
| A5 | Activin A | 0 | 0 | 0 | 0 |
| A6 | Activin A | 0.107 | 0 | 0 | 0 |
| A7 | ADAMTS-1 | 0.527 | 0.366 | 0 | 0.071 |
| A8 | ADAMTS-1 | 0.67 | 0.545 | 0.08 | 0 |
| A9 | Angiogenin | 1 | 0.768 | 9.152 | 41.438 |
| A10 | Angiogenin | 0 | 1.473 | 12.643 | 45.321 |
| A11 | Angiopoietin-1 | 5.223 | 25.661 | 48.527 | 88.786 |
| A12 | Angiopoietin-1 | 5.554 | 28.661 | 54.616 | 97.33 |
| A13 | Angiopoietin-2 | 63.786 | 38.92 | 34.027 | 23.107 |
| A14 | Angiopoietin-2 | 66.295 | 39.223 | 37.321 | 17.089 |
| A15 | Angiostatin / Plasminogen | 1.58 | 0 | 0.062 | 0 |
| A16 | Angiostatin / Plasminogen | 1.277 | 0 | 0 | 0 |
| A17 | Amphiregulin | 0.67 | 0 | 0 | 0 |
| A18 | Amphiregulin | 0 | 0 | 0 | 0 |
| A19 | Artemin | 11.714 | 4.116 | 0.955 | 2.286 |
| A20 | Artemin | 11.438 | 3.17 | 1.491 | 2.143 |
| A23 | Ref spots upper right | 242.679 | 237.08 | 244.179 | 248.375 |
| A24 | Ref spots upper right | 242.42 | 238.214 | 250.312 | 247.705 |
| B1 | Coagulation factor III | 1.143 | 0.536 | 0 | 0 |
| B2 | Coagulation factor III | 1.036 | 0.018 | 0 | 0 |
| B3 | CXCL16 | 0 | 0 | 0 | 0 |
| B4 | CXCL16 | 0 | 0 | 0 | 0 |
| B5 | DPPIV | 0 | 0 | 0 | 0 |
| B6 | DPPIV | 0 | 0 | 0 | 0.179 |
| B7 | EGF | 247.277 | 251.866 | 248.58 | 247.411 |
| B8 | EGF | 248.973 | 241.33 | 251.321 | 251.804 |
| B9 | EG-VEGF | 3.018 | 0 | 0 | 0 |
| B10 | EG-VEGF | 3.589 | 0.5 | 0 | 0 |
| B11 | Endoglin | 0 | 0 | 0 | 0 |
| B12 | Endoglin | 0.402 | 0 | 0 | 0 |
| B13 | Endostatin / Collagen XVIII | 24.491 | 6.08 | 0 | 0 |
| B14 | Endostatin / Collagen XVIII | 24.232 | 6.179 | 0.089 | 0 |
| B15 | Endothelin-1 | 212.089 | 179.955 | 81.339 | 22.741 |
| B16 | Endothelin-1 | 214.812 | 181.321 | 78.991 | 27.884 |
| B17 | FGF acidic | 0.616 | 0 | 0 | 0 |
| B18 | FGF acidic | 2.241 | 0 | 0 | 0 |
| B19 | FGF basic | 173.143 | 134.643 | 130.259 | 130.946 |
| B20 | FGF basic | 174.205 | 145.348 | 137.018 | 144.223 |
| B21 | FGF-4 | 0 | 0 | 0 | 0 |
| B22 | FGF-4 | 0 | 0 | 0 | 0 |
| B23 | FGF-7 | 0.589 | 0 | 0.071 | 0.107 |
| B24 | FGF-7 | 0.393 | 0 | 0.071 | 0.312 |
| C1 | GDNF | 0 | 0 | 0 | 0 |
| C2 | GDNF | 0 | 0 | 0 | 0 |
| C3 | GM-CSF | 0 | 0 | 0 | 0 |
| C4 | GM-CSF | 0 | 0 | 0.054 | 0 |
| C5 | HB-EGF | 0 | 0 | 0 | 0 |
| C6 | HB-EGF | 0.304 | 0 | 0 | 0 |
| C7 | HGF | 8.402 | 1.661 | 1.411 | 0.42 |
| C8 | HGF | 12.134 | 1.679 | 1.491 | 0.634 |
| C9 | IGFBP-1 | 25.277 | 8.973 | 10.214 | 19.732 |
| C10 | IGFBP-1 | 28.455 | 13.875 | 12.946 | 20.938 |
| C11 | IGFBP-2 | 7.259 | 7.268 | 10.562 | 24.929 |
| C12 | IGFBP-2 | 6.438 | 7.446 | 9.071 | 28.089 |
| C13 | IGFBP-3 | 0 | 0 | 0.071 | 0.036 |
| C14 | IGFBP-3 | 0.036 | 0 | 0.17 | 0.045 |
| C15 | IL-1ß | 6.598 | 0.161 | 0.161 | 0.009 |
| C16 | IL-1ß | 8.571 | 0.17 | 0.018 | 0 |
| C17 | IL-8 | 140.312 | 138.089 | 137.688 | 163.545 |
| C18 | IL-8 | 136.384 | 152.768 | 142.857 | 170.714 |
| C19 | LAP (TGF-ß1) | 0.625 | 0.589 | 0.134 | 1.25 |
| C20 | LAP (TGF-ß1) | 0.214 | 0.571 | 0.134 | 1 |
| C21 | Leptin | 0 | 0 | 0 | 0 |
| C22 | Leptin | 0 | 0 | 0 | 0 |
| C23 | MCP-1 | 0 | 0 | 0 | 0 |
| C24 | MCP-1 | 0 | 0 | 0 | 0 |
| D1 | MIP-1a | 0 | 0 | 0 | 0 |
| D2 | MIP-1a | 0 | 0 | 0 | 0 |
| D3 | MMP-8 | 2.741 | 0.17 | 0.33 | 0.009 |
| D4 | MMP-8 | 5.375 | 0.17 | 0.384 | 0.009 |
| D5 | MMP-9 | 0 | 0 | 0.241 | 0.045 |
| D6 | MMP-9 | 0 | 0 | 0.098 | 0 |
| D7 | NRG1-ß1 | 0 | 0.045 | 0.464 | 0.696 |
| D8 | NRG1-ß1 | 0.116 | 0.018 | 0.911 | 0.223 |
| D9 | Pentraxin 3 (PTX3) | 223.188 | 209.679 | 217.518 | 205.295 |
| D10 | Pentraxin 3 (PTX3) | 199.643 | 217.393 | 218.223 | 202.866 |
| D11 | PD-ECGF | 16.705 | 9.705 | 6.08 | 6.446 |
| D12 | PD-ECGF | 10.848 | 6.375 | 4.848 | 4.098 |
| D13 | PDGF-AA | 0.955 | 9.143 | 18.357 | 42.652 |
| D14 | PDGF-AA | 0.571 | 8.045 | 19.429 | 47.571 |
| D15 | PDGF-AB / PDGF-BB | 1 | 0.839 | 5.527 | 23.464 |
| D16 | PDGF-AB / PDGF-BB | 0 | 0.536 | 3.393 | 25.286 |
| D17 | Persephin | 2.58 | 0.357 | 0.625 | 0.17 |
| D18 | Persephin | 3.152 | 0.473 | 0.259 | 1.17 |
| D19 | Platelet Factor 4 (PF4) | 0.357 | 197.938 | 195.393 | 200.268 |
| D20 | Platelet Factor 4 (PF4) | 0 | 200.598 | 199.696 | 196.982 |
| D21 | PIGF | 1.607 | 0 | 0 | 0 |
| D22 | PIGF | 0.696 | 0 | 0 | 0 |
| D23 | Prolactin | 0 | 0 | 0 | 0 |
| D24 | Prolactin | 0 | 0 | 0 | 0 |
| E1 | Serpin B5 | 0.009 | 0 | 0 | 0 |
| E2 | Serpin B5 | 0.071 | 0.018 | 0 | 0 |
| E3 | Serpin E1 | 242.67 | 231.964 | 239.545 | 236.58 |
| E4 | Serpin E1 | 242.277 | 240.768 | 234.402 | 237.92 |
| E5 | Serpin F1 | 1 | 46.366 | 112.196 | 161.268 |
| E6 | Serpin F1 | 0 | 53.179 | 111.759 | 162.812 |
| E7 | TIMP-1 | 158.991 | 209.393 | 233.17 | 238.857 |
| E8 | TIMP-1 | 174.92 | 205.616 | 237.554 | 237.321 |
| E9 | TIMP-4 | 3.134 | 0 | 0 | 0.134 |
| E10 | TIMP-4 | 8.143 | 1.429 | 0.571 | 0.634 |
| E11 | Thrombospondin-1 | 248.062 | 240.098 | 239.188 | 231.661 |
| E12 | Thrombospondin-1 | 246.062 | 238.509 | 238.911 | 235.964 |
| E13 | Thrombospondin-2 | 2.661 | 0 | 0.036 | 0 |
| E14 | Thrombospondin-2 | 2.339 | 0 | 0.036 | 0.098 |
| E15 | uPA | 1.688 | 0 | 0 | 0 |
| E16 | uPA | 0.571 | 0 | 0 | 0 |
| E17 | Vasohibin | 0 | 0 | 0 | 0 |
| E18 | Vasohibin | 0.116 | 0 | 0 | 0 |
| E19 | VEGF | 78.929 | 59.161 | 46.875 | 55.286 |
| E20 | VEGF | 75.286 | 60.116 | 49.964 | 54.241 |
| E21 | VEGF-C | 0 | 0 | 0 | 0 |
| E22 | VEGF-C | 0 | 0 | 0 | 0 |
| F1 | Ref spots lower left | 246.625 | 246.946 | 247.089 | 244.875 |
| F2 | Ref spots lower left | 247.393 | 246.027 | 247.071 | 245.67 |
| F23 | Negative controls | 0 | 0 | 0 | 0 |
| F24 | Negative controls | 0 | 0 | 0 | 0 |
