# Supplementary material for: Platelet-released growth factors promote pro-angiogenic effects in endothelial cells: a translational perspective on wound healing and regenerative medicine
Source: Stem Cell Res Ther. 2026 Aug 5;17:270. doi: 10.1186/s13287-026-05214-y (PMC13445927; doi:10.1186/s13287-026-05214-y)
Supplement: Supplementary file 3 [file 13287_2026_5214_MOESM3_ESM.docx]

**Supplementary Table S1**

Coordinates of the target proteins on the array membrane:

| **Coordinate** | **Target** | **Alternate Nomenclature** |
| --- | --- | --- |
| A1, A2 | Reference Spots | N/A |
| A5, A6 | Activin A | — |
| A7, A8 | ADAMTS-1 | — |
| A9, A10 | Angiogenin | ANG |
| A11, A12 | Angiopoietin-1 | Ang-1 |
| A13, A14 | Angiopoietin-2 | Ang-2 |
| A15, A16 | Angiostatin/Plasminogen | — |
| A17, A18 | Amphiregulin | AR |
| A19, A20 | Artemin | — |
| A23, A24 | Reference Spots | N/A |
| B1, B2 | Coagulation Factor III | TF |
| B3, B4 | CXCL16 | — |
| B5, B6 | DPPIV | CD26 |
| B7, B8 | EGF | — |
| B9, B10 | EG-VEGF | PK1 |
| B11, B12 | Endoglin | CD105 |
| B13, B14 | Endostatin/Collagen XVIII | — |
| B15, B16 | Endothelin-1 | ET-1 |
| B17, B18 | FGF acidic | FGF-1 |
| B19, B20 | FGF basic | FGF2 |
| B21, B22 | FGF-4 | — |
| B23, B24 | FGF-7 | KGF |
| C1, C2 | GDNF | — |
| C3, C4 | GM-CSF | — |
| C5, C6 | HB-EGF | — |
| C7, C8 | HGF | — |
| C9, C10 | IGFBP-1 | — |
| C11, C12 | IGFBP-2 | — |
| C13, C14 | IGFBP-3 | — |
| C15, C16 | IL-1β | IL-1F2 |
| C17, C18 | IL-8 | CXCL8 |
| C19, C20 | LAP (TGF-β1) | — |
| C21, C22 | Leptin | — |
| C23, C24 | MCP-1 | CCL2 |
| D1, D2 | MIP-1α | CCL3 |
| D3, D4 | MMP-8 | — |
| D5, D6 | MMP-9 | — |
| D7, D8 | NRG1-β1 | HRG1-β1 |
| D9, D10 | Pentraxin 3 (PTX3) | TSG-14 |
| D11, D12 | PD-ECGF | — |
| D13, D14 | PDGF-AA | — |
| D15, D16 | PDGF-AB/PDGF-BB | — |
| D17, D18 | Persephin | — |
| D19, D20 | Platelet Factor 4 (PF4) | CXCL4 |
| D21, D22 | PlGF | — |
| D23, D24 | Prolactin | — |
| E1, E2 | Serpin B5 | Maspin |
| E3, E4 | Serpin E1 | PAI-1 |
| E5, E6 | Serpin F1 | PEDF |
| E7, E8 | TIMP-1 | — |
| E9, E10 | TIMP-4 | — |
| E11, E12 | Thrombospondin-1 | TSP-1 |
| E13, E14 | Thrombospondin-2 | TSP-2 |
| E15, E16 | uPA | — |
| E17, E18 | Vasohibin | — |
| E19, E20 | VEGF | — |
| E21, E22 | VEGF-C | — |
| F1, F2 | Reference Spots | N/A |
| F23, F24 | Negative Control | Control (-) |
