# Supplementary material for: Platelet-released growth factors promote pro-angiogenic effects in endothelial cells: a translational perspective on wound healing and regenerative medicine
Source: Stem Cell Res Ther. 2026 Aug 5;17:270. doi: 10.1186/s13287-026-05214-y (PMC13445927; doi:10.1186/s13287-026-05214-y)
Supplement: Supplementary file 4 [file 13287_2026_5214_MOESM4_ESM.docx]

**Supplementary Materials S4**

qPCR conditions and primers

qPCR was performed using TB Green Premix Ex Taq II (Takara Bio, Shiga, Japan) on a StepOnePlus instrument (Applied Biosystems, ThermoFisher, Dreieich, Germany) [1]. Cycling was done using the manufacturer-recommended shuttle PCR protocol: initial denaturation at 95 °C for 30 s (1 cycle), followed by 40 cycles of denaturation at 95 °C for 5 s and annealing/extension at 60 °C for 30 s. Serial cDNA dilutions were used to generate gene-specific standard curves. Expression was normalized to RP38. Primer sequences are listed below:

| **Gene** | **Forward Primer** | **Reverse Primer** |
| --- | --- | --- |
| ICAM-1 | ACCATCTACAGCTTTCCGG | ACACTTCACTGTCACCTCG |
| VCAM-1 | GGGAAGATGGTCGTGATCCTT | TCTGGGGTGGTCTCGATTTTA |
| IL-8 | ATTTCTGCAGCTCTGTGTGA | TCTCAGCCCTCTTCAAAAACT |
| ANG-2 | CTACGTGTCCAATGCTGTGC | GTTTGCTCCGCTGTTTGGTT |
| RP38 | TCAAGGACTTCCTGCTCACA | AAAGGTATCTGCTGCATCGAA |

**References:**

1. Roth SA, Simanski M, Rademacher F, Schröder L, Harder J. The Pattern Recognition Receptor NOD2 Mediates Staphylococcus aureus–Induced IL-17C Expression in Keratinocytes. Journal of Investigative Dermatology. Elsevier; 2014;134:374–80. https://doi.org/10.1038/JID.2013.313
