# Supplementary material for: Platelet-released growth factors promote pro-angiogenic effects in endothelial cells: a translational perspective on wound healing and regenerative medicine
Source: Stem Cell Res Ther. 2026 Aug 5;17:270. doi: 10.1186/s13287-026-05214-y (PMC13445927; doi:10.1186/s13287-026-05214-y)
Supplement: Supplementary file 5 [file 13287_2026_5214_MOESM5_ESM.docx]

**Supplementary Materials S2**

HUVEC culture and PRGF stimulation

All donors provided written informed consent in accordance with approval of the Ethics Committee of the University Medical Centre Schleswig-Holstein, Kiel, Germany (protocols D519/18; D518/13). HUVEC were isolated as previously described [1]. HUVEC were cultured in ECGM (PromoCell, Heidelberg, Germany) supplemented with 4 µL/mL endothelial cell growth supplement, 0.1 ng/mL EGF, 1 ng/mL bFGF, 90 µg/mL heparin, 1 µg/mL hydrocortisone (all PromoCell), and 10% FBS (Thermo Fisher, Dreieich, Germany) in a humidified incubator (37 °C, 5% CO₂). Cells were seeded at 2.4 × 10⁴ cells/cm² in 12-well plates (Sarstedt, Germany) and cultured until ~80% confluence. Medium was removed and replaced with PRGF at 2%, 5%, or 10% in ECGM, while ECGM without PRGF served as control. After 24h, supernatants were collected and stored at −20 °C for proteome profiling, while cells were processed for RNA extraction. Importantly, RNA and conditioned supernatants were obtained from the same wells and the same 24 h stimulation time point, ensuring direct comparability of transcriptional and secreted protein readouts.

**References:**

1. Baudin B, Bruneel A, Bosselut N, Vaubourdolle M. A protocol for isolation and culture of human umbilical vein endothelial cells. Nat Protoc. 2007;2:481–5. https://doi.org/10.1038/nprot.2007.54
