# Supplementary material for: Platelet-released growth factors promote pro-angiogenic effects in endothelial cells: a translational perspective on wound healing and regenerative medicine
Source: Stem Cell Res Ther. 2026 Aug 5;17:270. doi: 10.1186/s13287-026-05214-y (PMC13445927; doi:10.1186/s13287-026-05214-y)
Supplement: Supplementary file 6 [file 13287_2026_5214_MOESM6_ESM.docx]

**Supplementary Materials S3**

RNA isolation and cDNA synthesis

Total RNA was isolated using the RNeasy Mini Kit (Qiagen, Hilden, Germany) according to the manufacturer’s instructions. RNA quantity and purity were assessed by NanoDrop (ThermoFisher) and RNA was stored at −80 °C. cDNA synthesis was performed as previously described [1].

**References:**

1. Bayer A, Lammel J, Rademacher F, Groß J, Siggelkow M, Lippross S, et al. Platelet-released growth factors induce the antimicrobial peptide human beta-defensin-2 in primary keratinocytes. Exp Dermatol; 2016;25:460–5. https://doi.org/10.1111/EXD.12966
