# Supplementary material for: Platelet-released growth factors promote pro-angiogenic effects in endothelial cells: a translational perspective on wound healing and regenerative medicine
Source: Stem Cell Res Ther. 2026 Aug 5;17:270. doi: 10.1186/s13287-026-05214-y (PMC13445927; doi:10.1186/s13287-026-05214-y)
Supplement: Supplementary file 7 [file 13287_2026_5214_MOESM7_ESM.docx]

**Supplementary Materials S7**

VEGF and fibrinogen quantification; assessment of pro-angiogenic effects of PRGF fractions in OEC monoculture and OEC–ASC co-culture models:

PRGF was analysed for its VEGF content using VEGF ELISAs (R&D Systems Inc., Minneapolis, USA) in samples from individual donors or in samples, which were pooled for cell culture experiments. Further, the content of fibrinogen was assessed in the supernatant or the pellet of the PRGF samples centrifuged for 10.000 g for 20 minutes and 4 °C, followed by ELISAs for fibrinogen (Cusabio, Texas, USA) in accordance to the protocol by the manufacturer. For ELISAs from pellets of PRGF, samples were resuspended in PBS, frozen in liquid nitrogen and defrosted again for 3 times. The supernatants from these samples were collected after centrifugation at 5.000g for 5 minutes followed by ELISA.

The impact of the PRGF or PRGF derived fractions after centrifugation on the vascularization process was assessed using outgrowth endothelial cells (OEC) in monoculture, as well as in co-cultures of OEC and adipose tissue derived mesenchymal stromal cells (ASC), serving as a wound healing model. For this purpose, OEC were isolated and cultured as reported earlier from buffy coats [1,2]. For OEC mono-cultures cells were seeded on ibidi slides at 40.000 cells/well in EGM-2 supplemented with PRGF. For co-cultures ASC (Promocell, Heidelberg, Germany) were seed on 8 well ibidi slides in a density of 40.000 cells/well using MGM2 medium (Promocell, Heidelberg, Germany) supplemented with 10 % FCS. On the next day OEC were added to ASC at a density of 40.000 cells/well in EGM-2 (Lonza, Vervier, Belgium) supplemented with PRGF from the supernatant (vol %) or the pellet (mg/ml) as indicated in the experiments. To assess the formation of microvessel-like structures, mono-cultures or co-cultures were fixed after 7 days of culture and subjected to immunofluorescence staining for endothelial marker VE- cadherin and fibrin or smooth muscle actin, as well as nuclear staining.

Results S7: VEGF and fibrinogen quantification; assessment of pro-angiogenic effects of PRGF fractions in OEC monoculture and OEC–ASC co-culture models

To assess the functional impact of PRGF on angiogenesis in a wound healing scenario first mono-cultures of outgrowth endothelial cells (OEC) were treated with EGM-2 medium supplemented with PRGF. In media supplemented with 20% PRGF no significant VEGF levels were detected (Figure S7.1A). The initial concentrations in the media were below 50-100 ng/ml, typically applied in angiogenesis induction assays. VEGF levels, determined from OEC derived cell culture supernatants during the culture, were even below the detection limits (data not shown).

However, in OEC mono-cultures accumulations of matrix components were evident after 7 days, which were identified in immunofluorescence stainings as fibrin rich matrix complexes. Endothelial cells in monocultures formed micro-vessel-like structures along these fibrin rich areas (Figure S7.1B).

**Figure S7.1:** A. VEGF levels in EGM-2 supplemented with 20% PRGF detected by ELISA. B. Monocultures of OEC grown in EGM-2 or EGM-2 with 20% PRGF stained for vascular endothelial cadherin (VE cadherin, green; nuclear counterstain Hoechst, blue) and fibrin (depicted in red) after 7 days of culture. Scale bar 100µm.

Similar observations were obtained in co-cultures of OEC with adipose tissue derived mesenchymal stromal cells (ASC) used as an in-vitro model for a wound healing scenario. In co-cultures angiogenic structures were increased by supplementation with 20% PRGF and angiogenic structures were again associated with fibrin rich areas (Figure S7.2).

To further investigate the underlying mechanisms, PRGF samples were subjected to additional centrifugation to enrich high–molecular-weight pro-angiogenic components. The co-cultures were then treated with EGM-2 supplemented either with the PRGF supernatant or with the corresponding pelleted PRGF fractions. Notably, vascular structure formation was significantly enhanced in cultures treated with the pelleted PRGF fractions compared with those receiving the supernatant. These findings suggest that, in addition to soluble factors, high–molecular-weight proteins/molecules or complexes present in the pellet fraction and fibrin rich areas substantially contribute to the pro-angiogenic activity of PRGF (Figure S7.2).

**Figure S7.2:** Co-Cultures of OEC and ASC treated with different fractions of PRGF: 20% PRGF (total PRGF), 20% SN (20% supernatant after centrifugation in EGM-2), Pellet (10 mg pellet/ml EGM-2), Control (EGM-2). Endothelial cells were stained with VE Cadherin (depicted in red), samples were co-stained for fibrin or smooth muscle actin (ASC, depicted in green), nuclear counterstain (Hoechst, depicted in blue). Scale bar: 200 µm.

Further experiments indicated that fibrinogen is present in both PRGF supernatants and pellet fractions, with higher levels detected in the supernatants (Figure S7.3).

**Figure S7.3:** Assessment of soluble fibrinogen in PRGF fractions after centrifugation using ELISA. The fibrinogen content was assessed in the supernatant and in the pellet of centrifuged samples in pooled samples and in the 3 individual donor samples, ** p < 0.01; *** p < 0.001 paired t-test, n= 3 donors.

**Acknowledgements**

We thank Lennard Arp and Veit Hermann for their excellent experimental support.

**References:**

1. Fuchs S, Hermanns MI, Kirkpatrick CJ. Retention of a differentiated endothelial phenotype by outgrowth endothelial cells isolated from human peripheral blood and expanded in long-term cultures. Cell Tissue Res. 2006;326:79–92. https://doi.org/10.1007/s00441-006-0222-4

2. Kolbe M, Dohle E, Katerla D, Kirkpatrick CJ, Fuchs S. Enrichment of Outgrowth Endothelial Cells in High and Low Colony-Forming Cultures from Peripheral Blood Progenitors. Tissue Eng Part C Methods. 2010;16:877–86. https://doi.org/10.1089/ten.tec.2009.0492
