# Supplementary material for: Platelet-released growth factors promote pro-angiogenic effects in endothelial cells: a translational perspective on wound healing and regenerative medicine
Source: Stem Cell Res Ther. 2026 Aug 5;17:270. doi: 10.1186/s13287-026-05214-y (PMC13445927; doi:10.1186/s13287-026-05214-y)
Supplement: Supplementary file 8 [file 13287_2026_5214_MOESM8_ESM.docx]

**Supplementary Materials S6**

Endothelial Tube Formation Assay and Angiogenesis Analyzer Parameters

Tube formation assays were performed in ibidi µ-Slide 15 Well chambered coverslips (ibidi GmbH, Munich, Germany) using Corning® Matrigel® Matrix as extracellular matrix. Matrigel was handled on ice to prevent premature polymerization. All tubes, tips, and the slide were pre-cooled, Matrigel was dispensed slowly to minimize bubble formation. Wells containing visible air bubbles were excluded. After dispensing, Matrigel was polymerized at 37 °C before cell seeding. HUVEC (10.000 cells/well) were seeded onto the Matrigel layer in ECGM and treated with PRGF at 2%, 5%, or 10% concentration, while untreated cells served as control. Cultures were maintained at 37 °C, 5% CO₂ and phase-contrast images were acquired every hour for 8 h with an additional final image captured at 24 h. Tube formation was quantified using the ImageJ Angiogenesis Analyzer workflow [1] with identical analysis settings applied across all conditions; the readouts included morphometric parameters (e.g. segments, junctions, pieces, branches). Definitions of parameters and interpretation are provided below in the Supplementary Material.

**Definitions of Angiogenesis Analyzer parameters and their relevance for angiogenesis**

Definitions and interpretations of selected ImageJ Angiogenesis Analyzer outputs used for endothelial tube formation assays.

| **Parameter** | **Definition** | **Relevance for angiogenesis** | **P value (10% PRGF)** |
| --- | --- | --- | --- |
| Branching interval | Average distance between two successive branching points along a tube segment. | Shorter intervals indicate more frequent branching and a denser, more complex network; longer intervals suggest less branching and a simpler network. | ns |
| Tot. isol. branches length | Sum of the lengths of all isolated branches in the analyzed area (structures not connected to the main network). | Higher values suggest inefficient or incomplete tube formation, with structures failing to integrate into an interconnected network. | ns |
| Tot. branches length | Sum of the lengths of all individual branch segments within the network. | Higher values indicate more extensive branching and a more complex network, consistent with robust angiogenesis. | 0.0136 |
| Nb extremities | Total count of free ends/endpoints in the network. | Higher values can indicate incomplete connectivity, fragmentation, or a less organized network; lower values are typical of more interconnected networks. | 0.0133 |
| Tot. branching length | Total cumulative length of segments contributing to branching structures. | Higher values reflect a more extensive branching process that supports broader vascular coverage. | 0.0208 |
| Nb branches | Count of individual branch segments within the network. | Higher values indicate more branching events and a more intricate, expansive network. | 0.0275 |
| Tot. segments length | Sum of lengths of all tube segments (branching and non-branching). | Higher values indicate more extensive formation of vessel-like structures and pro-angiogenic activity. | ns |
| Tot. length | Sum of the lengths of all detected vascular-like structures in the analyzed field (overall network length). | Higher values indicate a larger extent of tube formation and network growth and provide a good representation of low-developed networks and tree-like structures in early tube-formation phenotypes. | 0.0135 |
| Nb isol. seg | Count of segments that are formed but not connected to the main network (standalone segments). | Higher values suggest inefficient or incomplete network formation, with poor integration into a functional vascular-like network. | ns |
| Nb segments | Total count of all individual tube segments identified in the network. | Higher values generally reflect greater network complexity but should be interpreted alongside connectivity-related metrics. | 0.0075 |
| Tot. master segments length | Cumulative length of primary (‘master’) tube structures from which branches extend. | Higher values suggest more developed core vessel-like structures forming the network backbone. | ns |
| Nb master segments | Count of primary (‘master’) tube structures. | Higher values indicate greater initiation of main vessel-like structures contributing to the angiogenic response. | 0.0108 |
| Mean mesh size | Average area of enclosed loops (‘meshes’) formed by interconnected segments. | Smaller mean mesh size typically reflects a denser network; larger meshes may indicate a looser or less mature structure. | ns |
| Mesh index | Derived measure combining mesh number and size to reflect network organization/complexity. | Provides an integrated view of network connectivity and organization; interpretation depends on how mesh number and size change together. | 0.0058 |
| Tot meshes area | Sum of areas of all meshes/loops in the network. | Higher values generally indicate a more extensive interconnected network and advanced morphogenesis. | ns |
| Nb meshes | Count of closed loops/enclosed areas formed by interconnected segments. | Higher values indicate a more complete and interconnected network, a key feature of functional angiogenesis in vitro. | 0.0177 |
| Nb master junction | Count of major connection points where master segments or key branches converge. | Higher values suggest more robust major connectivity and structural integrity of the network. | 0.0025 |
| Nb junctions | Total count of all junctions where two or more segments connect. | Higher values indicate a more branched and interconnected network, consistent with pro-angiogenic activity. | 0.0037 |
| Nb nodes | Count of nodes (general connection/branching points) in the network. | Higher values reflect greater network complexity and density. | 0.0031 |
| Nb pieces | Refers to the sum of all segments, isolated elements, and branches | Higher values indicate increased network complexity and branching activity and are often observed under pro-angiogenic conditions | 0.00049 |

ns = not significant; significant p-values (green = significant, red = ns); significance levels: *p < 0.05, **p < 0.01, **p < 0.001

**Reference:**

1. Carpentier G, Berndt S, Ferratge S, Rasband W, Cuendet M, Uzan G, et al. Angiogenesis Analyzer for ImageJ — A comparative morphometric analysis of “Endothelial Tube Formation Assay” and “Fibrin Bead Assay.” Sci Rep. 2020;10:11568. https://doi.org/10.1038/s41598-020-67289-8
