# Supplementary material for: Platelet-released growth factors promote pro-angiogenic effects in endothelial cells: a translational perspective on wound healing and regenerative medicine
Source: Stem Cell Res Ther. 2026 Aug 5;17:270. doi: 10.1186/s13287-026-05214-y (PMC13445927; doi:10.1186/s13287-026-05214-y)
Supplement: Supplementary file 9 [file 13287_2026_5214_MOESM9_ESM.docx]

**Supplementary Materials S5**

Human Angiogenesis Proteome Profiler Array

Supernatants from PRGF-treated (2%, 5%, 10%; 24 h) HUVEC and untreated controls were analyzed using the Proteome Profiler Human Angiogenesis Array Kit (#ARY007, R&D Systems, Minneapolis, USA) following the manufacturer’s protocol. The membranes include internal reference spots and negative control spots for assay verification.

To support accurate attribution in PRGF-conditioned experiments, two baseline reference profiles were processed under identical assay conditions: BL1 (ECGM, no cell contact) to capture culture-medium background signals, and BL2 (10% PRGF in PBS, no cell contact) to account for proteins intrinsically present in PRGF. BL2 was generated using representative donor-derived PRGF preparations processed identically to the main experiments.

Briefly, nitrocellulose membranes were equilibrated in B7 (2 mL; 1 h; RT; shaking). For each membrane, the sample mixture (SM) was prepared by combining 700 µL sample with B4 (500 µL) and B5 (300 µL); DAC (15 µL) was added and incubated for 1 h at RT. After removal of B7, 700 µL SM was applied to each membrane and incubated overnight at 4 °C on a shaking platform. Membranes were washed with WB (20 mL per wash; 3 × 10 min), incubated with SA-HRP (2 mL; 30 min; RT; rocking), and washed again with WB (20 mL per wash; 3 × 10 min). For detection, membranes were incubated with CR (1 mL; 1 min) bubble-free between plastic sheets. Chemiluminescence was documented using the Fusion FX system (Vilber) and quantified by densitometry (ImageJ/Vilber). Targets were considered for interpretation if signal intensity exceeded >10% of reference-spot intensity. Outputs for all 55 targets across all conditions are provided in Supplementary Table S2_Signal Value.

To support reproducibility, the array readout was replicated under identical conditions.

| **Abbreviation** | **Kit component** | **Use in workflow** | **Amount / condition** |
| --- | --- | --- | --- |
| B7 | Array Buffer 7 | Membrane blocking/equilibration | 2 mL; 1 h; RT; shaking |
| B4 | Array Buffer 4 | Sample mixture (diluent) | 500 µL |
| B5 | Array Buffer 5 | Sample mixture (diluent) | 300 µL |
| DAC | Detection Antibody Cocktail | Sample pre-incubation | 15 µL; 1 h; RT |
| SM | Sample mixture (Sample + B4 + B5 + DAC) | Incubation on membrane | 700 µL; overnight; 4 °C; shaking |
| WB | Wash Buffer | Washing steps | 20 mL per wash; 3×10 min after SM; 3×10 min after SA-HRP |
| SA-HRP | Streptavidin–HRP | Detection incubation | 2 mL; 30 min; RT; shaking |
| CR | Chemi Reagent Mix | Chemiluminescent development | 1 mL; 1 min; bubble-free between plastic sheets |
| BL1 | ECGM without HUVEC contact | Baseline reference: medium background | Processed in parallel, identical workflow |
| BL2 | PBS + PRGF without HUVEC | Baseline reference: PRGF-derived proteins | Processed in parallel, identical workflow |
